# The impact of corneal higher-order aberrations on dynamic visual acuity post cataract surgery

**DOI:** 10.3389/fnins.2024.1321423

**Published:** 2024-05-13

**Authors:** Tingyi Wu, Yuexin Wang, Yuanting Li, Yuanhong Li, Xiaodan Jiang, Xuemin Li

**Affiliations:** ^1^Department of Ophthalmology, Peking University Third Hospital, Beijing, China; ^2^Beijing Key Laboratory of Restoration of Damaged Ocular Nerve, Peking University Third Hospital, Beijing, China

**Keywords:** dynamic visual acuity (DVA), dynamic visual acuity test (DVAT), dynamic visual function, corneal higher-order aberrations, cataract

## Abstract

**Purpose:**

To explore the influence of corneal higher-order aberrations (HOAs) on dynamic visual acuity (DVA) post cataract surgery.

**Methods:**

A total of 27 patients with 45 eyes following cataract surgery were included in this study. The postoperative monocular object-moving DVA at the velocity of 20, 40, and 80 degrees per second (dps) were examined at 1 month. The total corneal HOAs were measured with Scheimpflug-based corneal topography. The correlation between postoperative DVA and HOAs was analyzed.

**Results:**

Significant difference was shown among DVA at different velocities (*P* < 0.001). The 20 dps DVA was significantly better than 40 (*P* < 0.001) and 80 (*P* < 0.001) dps DVA. No significant difference was observed between 40 and 80 dps DVA (*P* = 0.420). The vertical coma and the root mean square (RMS) of coma (RMS_*coma*_) were statistically correlated with 80 dps DVA (*P* < 0.05). The vertical trefoil, RMS_*trefoil*_ and total RMS_*HOA*_ were statistically correlated with 40 and 80 dps DVA (*P* < 0.05). The spherical aberration was not significantly associated with postoperative DVA (*P* > 0.05 for all velocites). The multivariate linear regression model revealed that age was a significant influential factor for 20 dps DVA (*P* = 0.002), and RMS_*trefoil*_ (4 mm) and age were significantly associated with 40 and 80 dps DVA (*P* ≤ 0.01).

**Conclusion:**

The research demonstrated that larger corneal HOAs, especially coma and trefoil aberrations were significantly associated with worse high-speed DVA, but not spherical aberration post cataract surgery.

## Introduction

Cataract is a prevalent ocular disease causing blindness in elderly patients (Hashemi et al., 2020). It is corroborated that early surgical intervention is associated with superior quality of life in cataract patients (Assi et al., 2021). Currently, modern cataract surgery stresses improving postoperative visual function in daily scenarios (Hashemi et al., 2020; Jing et al., 2022). Real-life visual function cannot be sufficiently evaluated by static visual acuity (VA). Postoperative patients require functional vision to catch moving objects in daily tasks, including sports and driving (Steinberg et al., 1994). Dynamic visual function, crucial in daily activities, has been investigated in several previous research (Ao et al., 2014; Ren et al., 2020; Ke et al., 2022; Wang et al., 2023; Wu et al., 2023). Dynamic visual acuity (DVA) test evaluates the ability to identify the details of moving visual targets, and it can assess the cataract surgical outcomes more sensitively compared with the static VA test (Ao et al., 2014). Significant improvement in DVA was observed after cataract surgery (Ren et al., 2020).

An ideal optical system converges external light rays into a focal point on the fovea to create a clear image. However, deviation exists between the ideal and actual wavefront in human eyes, called aberrations (Oliveira et al., 2012). Optical aberrations can be divided into higher-order ones (HOAs) and lower-order ones (LOAs). LOAs, which contain defocus and regular astigmatism, account for nearly 90% of the total aberrations in human eyes and can be corrected with spectacles or contact lenses (Lombardo and Lombardo, 2010). HOAs, such as coma and spherical aberrations, cannot be corrected with conventional methods (Maloney et al., 1993). HOAs can degrade image quality, though they contribute far less to the total ocular wavefront aberrations than LOAs (Lombardo and Lombardo, 2010).

Corneal aberrations constitute the fundamental part of total ocular aberrations (Ye et al., 2014), and are crucial to visual quality when the intraocular lens (IOL) is centered in pseudophakic eyes. According to the study of Jing et al., there were no significant differences in best-corrected static VA 1 month and 3 months after cataract surgery, while corneal HOAs showed statistical differences (Jing et al., 2022). In the DVA test, subjects are required to trace the moving optotypes. Therefore, the dynamic visual performance may be more sensitive to visual disturbance created by corneal HOAs (Rocha et al., 2007). Although corneal HOAs have a limited effect on static visual performance, they may influence DVA, which remains to be explored. We aim to explore the influence of corneal HOAs on DVA in patients following uneven cataract surgery prospectively in this study. The present study might provide DVA-related task guidance to patients with various corneal HOAs after cataract surgery.

## Materials and methods

### Study design

This study was a single-center, prospective case series to investigate the impact of corneal HOAs on DVA in age-related cataract patients at Peking University Third Hospital. Written informed consent was obtained from each patient. The research was conducted in accordance with the ethical principles of the Declaration of Helsinki and was approved by the Ethics Committee of Peking University Third Hospital (NO. M2023201). The inclusion criteria were consecutive patients aged 50–80, diagnosed with age-related cataract and planning to receive phacoemulsification with intracapsular IOLs implantation targeting Plano. The exclusion criteria were as follows: (1) high corneal astigmatism (≥2.00 D); (2) severe ocular diseases, including keratoconus, corneal degeneration, glaucoma, uveitis, retinal diseases, eye alignment problems and extraocular movement problems; (3) history of intraocular surgeries or corneal refractive surgeries; (4) severe systemic diseases or cognitive disorders that may affect DVA; (5) severe intraoperative or postoperative complications, including posterior capsule rupture, zonule dialysis and endophthalmitis; (6) postoperative best corrected static VA > 0.1 logMAR.

The implanted IOL was DIFF-aA (HumanOptics Aktiengesellschaft, Erlangen, Germany). It is a one-piece biconvex hydrophilic acrylic diffractive bifocal IOL with a +3.5D near addition and no inherent spherical aberrations. This spherical aberration-free IOL has a central diffractive zone and a peripheral refractive zone, providing excellent vision in photopic and mesopic lighting conditions (Dexl et al., 2014; Schrecker et al., 2014).

### Preoperative evaluation

Preoperative evaluation included uncorrected distance VA measure (UDVA, 5 m, standard logMAR VA chart), intraocular pressure (noncontact tonometer, Nidek NT-530 software version 1.11, NIDEK Co., Ltd., Aichi, Japan), slit lamp biomicroscopy, indirect ophthalmoscopy, ocular biometry (IOL Master 700 software version 1.90.38.02, Carl Zeiss Meditec AG, Jena, Germany), corneal topography (Pentacam HR software version 1.21r43, OCULUS Optikgeräte GmbH, Wetzlar, Germany), optical coherence tomography (SPECTRALIS^®^ software version 6.12.4, Heidelberg Engineering GmbH, Heidelberg, Germany) and noncontact specular microscope (Nidek CEM-530 software version V1.15.01, NIDEK Co., Ltd., Aichi, Japan). The Barrett Universal II formula with a lens constant of 119.1 was used for IOL power calculation, and the IOL diopter with the corresponding refractive prediction closest to the Plano target in the monogram was selected.

### Surgical procedures

A 3.2 mm clear corneal main incision was made at 10 o’clock, and the assisted incision was made at 2 o’clock. Continuous curvilinear capsulorhexis 5 ∼ 5.5 mm in diameter was conducted. The balanced salt solution was used for hydrodissection and hydrodelineation. Subsequently, standard phacoemulsification was performed for nuclear fragmentation and extraction. Then, irrigation and aspiration (I/A) were applied to clear the cortex, followed by posterior capsule polishing. After injecting viscoelastic into the capsular bag, the IOL was implanted. The viscoelastic was removed with I/A, followed by stromal hydration for corneal incision sealing. After the surgery, 0.5% Cravit^®^ (Santen, Osaka, Japan) and 1% Pred Forte^®^ (Allergan, AbbVie Inc., North Chicago, IL, USA) were given four times daily for a month.

### Postoperative evaluation

Postoperative evaluation was performed 1 day, week and month after surgery. UDVA (5 m), slit lamp biomicroscopy and intraocular pressure (noncontact tonometer) were evaluated at each follow-up time point. At 1-month follow-up, uncorrected intermediate VA (UIVA, 80 cm), uncorrected near VA (UNVA, 40 cm), noncycloplegic subjective refraction, corneal HOAs and DVA were further assessed.

### Corneal higher-order aberrations

Corneal HOAs were measured using a Scheimpflug-based Pentacam HR system. The scan was conducted in a darkroom, and the measurement was regarded as valid data if the quality specification window demonstrated ‘OK’ (Xi, 2020). HOAs of 4-, 5-, 6-, and 7-mm analysis diameters were recorded. The outcomes were demonstrated by the Zernike polynomials. The root mean square (RMS, μm) of corneal total HOAs (RMS_HOA_, from third-order to sixth-order), coma aberrations (RMS_coma_, $Z_{3}^{-1}$, $Z_{3}^{1}$, $Z_{5}^{-1}$ and $Z_{5}^{1}$combined), trefoil aberrations (RMS_trefoil_, $Z_{3}^{-3}$, $Z_{3}^{3}$, $Z_{5}^{-3}$ and $Z_{5}^{3}$ combined) and spherical aberrations (RMS_sphere_, $Z_{4}^{0}$ and $Z_{6}^{0}$ combined) were calculated, and the equation is shown below:

$$R\,M\,S_{c\,o\,m\,a}=\sqrt{{\left(Z_{3}^{-1}\right)}^{2}+{\left(Z_{3}^{1}\right)}^{2}+{\left(Z_{5}^{-1}\right)}^{2}+{\left(Z_{5}^{1}\right)}^{2}}$$

$$R\,M\,S_{t\,r\,e\,f\,o\,i\,l}=\sqrt{{\left(Z_{3}^{-3}\right)}^{2}+{\left(Z_{3}^{3}\right)}^{2}+{\left(Z_{5}^{-3}\right)}^{2}+{\left(Z_{5}^{3}\right)}^{2}}$$

$$R\,M\,S_{s\,p\,h\,e\,r\,e}=\sqrt{{\left(Z_{4}^{0}\right)}^{2}+{\left(Z_{6}^{0}\right)}^{2}}$$

The Strehl ratio (SR) is the ratio of the point spread function between the measured eye and the ideal eye and is directly related to visual quality (Miháltz and Vécsei-Marlovits, 2021). It could be calculated as follows:

$$S\,R=e^{-{\left(\frac{2\,\pi }{\lambda }\,R\,M\,S\right)}^{2}}$$

where λ was calculated with a reference value of 555 nm, and RMS was the root mean square of corneal total aberrations (RMS_HOA_) (Xu et al., 2022).

### Dynamic visual acuity test

The self-developed program for the DVA test was run by MATLAB2017b (The MathWorks, Inc., Natick, MA, USA). A twisted nematic monitor (24 inches, interface DisplayPort 1.2, resolution 1920 × 1080, refresh rate 144 Hz, response time 1 ms, luminance 30 lux) was applied to display the moving optotypes. The dynamic optotypes were designed according to the standard logMAR VA chart. The program generated E optotypes moving horizontally from the left to the right in the middle of the monitor. The opening direction of the optotype was randomized at each display. The size and velocity can be adjusted by the examiner. DVA at three velocities was measured in this research, including 20, 40 and 80 degrees per second (dps). Patients were seated three meters away from the monitor with their eyes at the same height as the center of the monitor and required to identify the opening direction of the optotype. Ametropia was fully corrected with spectacles based on noncycloplegic subjective refraction during the test. The DVA test procedures were described in detail in our previous articles (Ao et al., 2014; Wu et al., 2022). The minimum size of the optotype that the patient could identify was recorded as logMAR.

### Statistical analysis

All statistical analyses were completed with SPSS (version 29.0, IBM Corp., Armonk, NY, USA). P ≤ 0.05 was set as statistically significant. Continuous variables were represented as mean ± SD. The normal distribution of the data was examined by the Kolmogorov-Smirnov test. The comparison between preoperative and postoperative static UDVA was accomplished by the Wilcoxon Signed-Ranks test. The comparison among postoperative static UDVA, UIVA, and UNVA was accomplished by the Friedman test. The comparison among postoperative corrected distance DVA under the velocity of 20, 40, and 80 dps was accomplished by the Friedman test. Bonferroni correction was used in post-hoc analysis. The correlation between DVA and HOAs (including $Z_{3}^{-1}$, $Z_{3}^{1}$, $Z_{3}^{-3}$, $Z_{3}^{3}$, $Z_{4}^{0}$, $Z_{5}^{-1}$, $Z_{5}^{1}$, $Z_{5}^{-3}$, $Z_{5}^{3}$, $Z_{6}^{0}$, RMS_HOA_, RMS_coma_, RMS_trefoil_, RMS_sphere_ and SR) were analyzed using Spearman correlation analysis with the resampling technique the Bootstrap adopted. A multivariate linear regression model was further established to analyze the associated factors for postoperative DVA.

## Results

A total of 27 patients with 45 eyes were enrolled in this study. The demographic parameters are summarized in Table 1. The mean age was 67.3 years old, and males accounted for 29.6% of the participants.

**TABLE 1:** Preoperative demographics.

Parameters	Mean ± SD	Range (min, max)
Age (years)	67.3 ± 6.5	54, 78
Corneal astigmatism (D)	0.61 ± 0.38	0, 1.84
Corneal endothelial cell count (/mm^2^)	2625 ± 388	1674, 3525
IOL power (D)	21.49 ± 1.95	17, 26
Target SE (D)	−0.03 ± 0.13	−0.31, 0.17
Axial length (mm)	23.25 ± 0.61	22.07, 24.31

D, diopter; IOL, intraocular lens; SD, standard deviation; SE, spherical equivalent.

### Visual acuity and refraction

**FIGURE 1:**
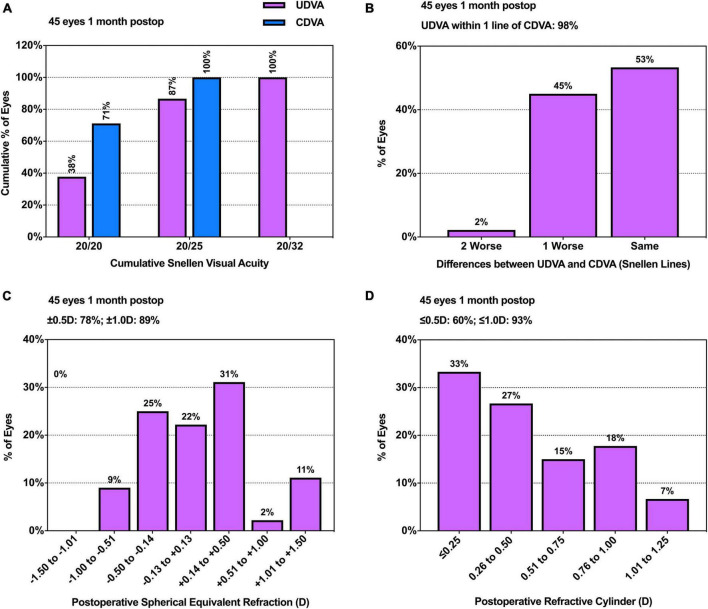
Histograms of postoperative static visual outcomes. **(A)** Uncorrected and corrected distance visual acuity; **(B)** Uncorrected versus corrected distance visual acuity; **(C)** Spherical equivalent refraction accuracy; **(D)** Postoperative refractive cylinder. CDVA, corrected distance visual acuity; postop, postoperative; UDVA, uncorrected distance visual acuity.

The graphs reporting postoperative static VA and refractive outcomes are shown in Figure 1. The results revealed that 87% of the included patients achieved 20/25 in Snellen UDVA. The UDVA within 1 line of CDVA was 98%. The spherical equivalent within ± 1.0D was 89% and the refractive cylinder ≤ 1.0D was 93%. The results of visual acuity and postoperative refraction are summarized in Table 2. The results indicated that the UDVA significantly improved after surgery (*Z* = −5.605, *P* < 0.001). The postoperative DVA at the velocity of 20, 40, and 80 dps was 0.326 ± 0.111, 0.544 ± 0.112 and 0.594 ± 0.158, respectively. A significant difference was shown among different velocities (χ^2^(2) = 73.345, *P* < 0.001). Pairwise comparison analysis revealed that 20 dps DVA was significantly better than 40 (*P* < 0.001) and 80 (*P* < 0.001) dps DVA. There was no significant difference between 40 and 80 dps DVA (*P* = 0.420).

**TABLE 2:** Visual acuity and postoperative refraction.

Parameters	Mean ± SD	Range (min, max)
**Preoperative static VA**		
UDVA (logMAR, 5 m)	0.452 ± 0.316	0, 1.854
**Postoperative static VA**		
UDVA (logMAR, 5 m)	0.076 ± 0.068	0, 0.200
CDVA (logMAR, 5 m)	0.029 ± 0.046	0, 0.100
UIVA (logMAR, 80 cm)	0.169 ± 0.112	0, 0.400
UNVA (logMAR, 40 cm)	0.296 ± 0.102	0.100, 0.600
**Postoperative corrected distance dynamic VA**		
20 dps (logMAR, 3 m)	0.326 ± 0.111	0.088, 0.588
40 dps (logMAR, 3 m)	0.544 ± 0.112	0.275, 0.875
80 dps (logMAR, 3 m)	0.594 ± 0.158	0.263, 1.050
**Postoperative refraction**		
Sphere (D)	0.378 ± 0.521	−0.750, 1.500
Cylinder (D)	−0.511 ± 0.423	−1.250, 0
Spherical equivalent (D)	0.122 ± 0.556	−0.750, 1.500

CDVA, corrected distance visual acuity; D, diopter; dps, degrees per second; SD, standard deviation; UDVA, uncorrected distance visual acuity; UIVA, uncorrected intermediate visual acuity; UNVA, uncorrected near visual acuity; VA, visual acuity.

### Correlation between DVA and corneal HOAs

The results of postoperative corneal HOAs are summarized in Table 3. The results of Spearman correlation analysis between postoperative DVA and RMS of corneal HOAs at each diameter are illustrated in Table 4. The correlation analysis demonstrated that the DVA at 40 and 80 dps was significantly correlated with the RMS of corneal total HOAs with 4-, 5-, 6- and 7-mm diameters (*P* < 0.01 for all analyses). In contrast, the 20 dps DVA was not significantly correlated with the RMS of corneal total HOAs (*P* > 0.05 for all analysis diameters). Among different types of HOAs, the DVA at 20, 40 or 80 dps was not associated with RMS_sphere_ in all analysis diameters (*P* > 0.05 for all analyses). The correlation analysis between DVA and coma aberrations revealed that 80 dps DVA was significantly positively correlated with RMS_coma_ in 4- (*P* = 0.034) and 5-mm (*P* = 0.023) analysis diameters. For trefoil aberration, the DVA at 40 and 80 dps was significantly positively correlated with RMS_trefoil_ in all analysis diameters (*P* < 0.01 for all analyses). The correlation analysis between corneal HOAs represented by the Zernike polynomial coefficient and DVA is demonstrated in the Supplementary Table 1. The correlation analysis between DVA and coma aberrations revealed that the 80 dps DVA was statistically positively correlated with $Z_{3}^{-1}$ in all analysis diameters (*P* < 0.05 for all analyses). There was a notable negative correlation between 40 dps DVA and $Z_{3}^{-3}$ in all analysis diameters (*P* < 0.05 for all analyses), as well as 80 dps DVA and $Z_{3}^{-3}$ in 5-, 6- and 7-mm analysis diameters (*P* < 0.05 for all analyses). A significant negative correlation was also observed between 20 dps DVA and $Z_{5}^{-3}$ in 6-mm analysis diameter (*P* = 0.037).

**TABLE 3:** Corneal higher-order aberrations.

	Analysis diameter			
**Parameters (μ m)**	**4 mm**	**5 mm**	**6 mm**	**7 mm**
Total HOA RMS	0.255 ± 0.096	0.494 ± 0.213	0.871 ± 0.382	1.426 ± 0.583
Strehl ratio^†^	0.019 ± 0.038	–	–	–
$Z_{4}^{0}$	0.065 ± 0.035	0.158 ± 0.065	0.319 ± 0.106	0.534 ± 0.173
$Z_{6}^{0}$	0.001 ± 0.009	0 ± 0.017	−0.005 ± 0.032	−0.037 ± 0.055
Spherical HOA RMS (RMS_sphere_)	0.066 ± 0.034	0.159 ± 0.064	0.321 ± 0.105	0.538 ± 0.173
$Z_{3}^{-1}$	0.038 ± 0.132	0.080 ± 0.224	0.114 ± 0.336	0.098 ± 0.458
$Z_{3}^{1}$	−0.007 ± 0.080	0.011 ± 0.112	0.045 ± 0.172	0.080 ± 0.339
$Z_{5}^{-1}$	0.006 ± 0.024	0.002 ± 0.041	−0.016 ± 0.062	−0.062 ± 0.104
$Z_{5}^{1}$	0.007 ± 0.020	0.013 ± 0.036	0.016 ± 0.059	0.009 ± 0.116
Coma HOA RMS (RMS_coma_)	0.145 ± 0.070	0.237 ± 0.123	0.357 ± 0.189	0.529 ± 0.289
$Z_{3}^{-3}$	−0.097 ± 0.108	−0.249 ± 0.215	−0.475 ± 0.364	−0.771 ± 0.533
$Z_{3}^{3}$	−0.019 ± 0.074	−0.027 ± 0.149	−0.037 ± 0.289	−0.058 ± 0.498
$Z_{5}^{-3}$	−0.020 ± 0.024	−0.031 ± 0.032	−0.031 ± 0.046	−0.018 ± 0.069
$Z_{5}^{3}$	0.003 ± 0.020	0.005 ± 0.032	0.007 ± 0.051	0.001 ± 0.075
Trefoil HOA RMS (RMS_trefoil_)	0.139 ± 0.093	0.305 ± 0.201	0.566 ± 0.354	0.925 ± 0.528

HOA, higher-order aberration; RMS, root mean square.

^†^We did not calculate the Strehl ratio under the analysis diameter of 5-,6- and 7-mm, because the values were too small with the increasing RMS_HOA_.

**TABLE 4:** Correlation analysis between postoperative dynamic visual acuity and root mean square of corneal higher-order aberrations.

Parameters	DVA (20 dps)		DVA (40 dps)		DVA (80 dps)	
	***R***	***P***	***R***	***P***	***R***	***P***
Total HOA RMS (4 mm)	0.232	0.126	0.508	<0.001**	0.451	0.002**
Total HOA RMS (5 mm)	0.244	0.106	0.476	<0.001**	0.471	0.001**
Total HOA RMS (6 mm)	0.212	0.162	0.415	0.005**	0.408	0.005**
Total HOA RMS (7 mm)	0.198	0.192	0.397	0.007**	0.402	0.006**
Strehl ratio (4 mm)	−0.232	0.126	−0.508	<0.001**	−0.451	0.002**
RMS_sphere_ (4 mm)	−0.123	0.420	0.045	0.770	0.126	0.408
RMS_sphere_ (5 mm)	−0.143	0.347	−0.042	0.786	0.112	0.462
RMS_sphere_ (6 mm)	−0.058	0.706	−0.077	0.616	0.098	0.523
RMS_sphere_ (7 mm)	−0.036	0.812	−0.097	0.525	0.034	0.824
RMS_coma_ (4 mm)	0.236	0.119	0.245	0.105	0.317	0.034*
RMS_coma_ (5 mm)	0.246	0.104	0.227	0.133	0.339	0.023*
RMS_coma_ (6 mm)	0.122	0.424	0.126	0.411	0.258	0.087
RMS_coma_ (7 mm)	−0.043	0.777	0.099	0.517	0.247	0.101
RMS_trefoil_ (4 mm)	0.052	0.735	0.412	0.005**	0.400	0.006**
RMS_trefoil_ (5 mm)	0.137	0.369	0.487	<0.001**	0.472	0.001**
RMS_trefoil_ (6 mm)	0.175	0.251	0.472	0.001**	0.431	0.003**
RMS_trefoil_ (7 mm)	0.197	0.195	0.429	0.003**	0.406	0.006**

Dps, degrees per second; DVA, dynamic visual acuity; HOA, higher-order aberration; P, probability value; R, correlation coefficient; RMS, root mean square. The boldface indicates statistical significance.

*Indicates statistical significance *P* ≤ 0.05.

**Indicates statistical significance *P* ≤ 0.01.

### Influential factor analysis for DVA

Multivariate linear regression was implemented to fit DVA. Considering the multicollinearity, age, RMS_sphere_ (4 mm), RMS_coma_ (4 mm) and RMS_trefoil_ (4 mm) were included in the model, and the results are demonstrated in Table 5. The variance inflation factors of all independent variables were smaller than 10. The results revealed that age was significantly positively associated with 20 dps (*P* = 0.002), 40 dps (*P* = 0.01) and 80 dps (*P* = 0.01) DVA. RMS_trefoil_ (4 mm) was significantly positively associated with 40 dps (*P* = 0.005) and 80 dps (*P* = 0.008) DVA. RMS_sphere_ and RMS_coma_ were not significantly associated with postoperative DVA (*P* > 0.05 for all the analyses).

**TABLE 5:** Multivariate linear regression analysis for dynamic visual acuity.

Dependent variables	Independent variables	Standardized coefficient β	*P*	R^2^
DVA (20 dps)	Age	0.480	0.002**	0.217
	RMS_sphere_ (4 mm)	−0.213	0.145	
	RMS_coma_(4 mm)	0.109	0.443	
	RMS_trefoil_ (4 mm)	0.087	0.555	
DVA (40 dps)	Age	0.365	0.010**	0.293
	RMS_sphere_ (4 mm)	−0.141	0.306	
	RMS_coma_(4 mm)	0.053	0.691	
	RMS_trefoil_ (4 mm)	0.411	0.005**	
DVA (80 dps)	Age	0.367	0.010**	0.283
	RMS_sphere_ (4 mm)	−0.093	0.501	
	RMS_coma_(4 mm)	0.053	0.694	
	RMS_trefoil_ (4 mm)	0.390	0.008**	

Dps, degrees per second; DVA, dynamic visual acuity; P, probability value; R, correlation coefficient; RMS, root mean square. The boldface indicates statistical significance.

**Indicates statistical significance *P* ≤ 0.01.

## Discussion

Current visual function evaluation for postoperative cataract patients mainly focuses on static vision. DVA is a promising parameter for functional vision. However, the influential factors for DVA in post-cataract surgery patients are not sufficiently understood. To the best of our knowledge, the present study is the first to investigate the influence of corneal HOAs on DVA post-cataract surgery, which provides potential guidance on DVA-related tasks for patients with different corneal HOAs.

The implantation of DIFF-aA provided favorable postoperative static visual outcomes in our study. All patients reached 0.2 or better, and 86.7% reached 0.1 or better UDVA. 73.3% of the patients reached 0.2 or better UIVA, and 75.6% reached 0.3 or better UNVA. Similar results in corneal HOAs were observed in a previous study analyzing the optical quality after implantation of DIFF-aA IOL, which were 0.22 ± 0.11 in RMS_HOA_, 0.11 ± 0.06 in coma aberration and −0.036 ± 0.052 in spherical aberration for 4 mm analysis diameter (Miháltz and Vécsei-Marlovits, 2021).

Our study found that the larger the vertical coma and RMS_coma_, the worse the dynamic visual performance under high velocity. Coma aberration causes multifocality along the meridian and creats trailing ‘comet-like’ blur (Nanavaty et al., 2010), which affects the trajectory judgment of moving objects and influences DVA. The influence of RMS_coma_ on 80 dps DVA was limited to the small pupil size, possibly due to the photopic DVA testing environment. Previous research also found that coma aberration significantly affected visual quality. In the study of Tabernero et al. (2007), the authors found that coma-correcting IOL provided better retinal image quality than conventional IOL. Yamaguchi et al. observed that coma aberration was negatively correlated with contrast sensitivity under photopic conditions (Yamaguchi et al., 2011). McCormick et al. found that the value of vertical coma was higher in patients with visual complaints compared with asymptomatic patients after laser *in situ* keratomileusis surgery (McCormick et al., 2005). Further analyzing the impact of different types of coma aberration, we found that vertical coma was significantly associated with DVA instead of horizontal coma. The dynamic optotypes were moving horizontally in our study. Thus, the vertical trailing images might impact the identification of moving objects more than the horizontal trailing images.

Our study found that the DVA at 40 and 80 dps was significantly correlated with the vertical trefoil and RMS_trefoil_. The schematic diagram of trefoil aberration demonstrates that it produces triangular astigmatism causing a light spot to smear in three directions (Sinjab, 2021). The star-like retinal image, induced by trefoil aberration, might influence the observance of moving objects in the direction overlapping the stelliform light. Thus, the DVA is affected. Previous studies also found that trefoil aberration affected visual quality. Fernández-Sánchez et al. found that trefoil aberration significantly reduced high-contrast VA, low-contrast VA and contrast sensitivity (Fernández-Sánchez et al., 2008). The study of Ishiguro et al. demonstrated a significant correlation between the RMS value of total trefoil and photophobia score in patients after cataract surgery (Ishiguro et al., 2022). Atchison et al. also mentioned that the increase in trefoil aberration could affect the contrast of letter images and cause blurry vision (Atchison et al., 2009).

The SR is an objective metric to evaluate retinal image quality. When the RMS is 0, the value of SR is 1, indicating perfect image quality. The value gradually declines to the minimum 0, along with the degradation of image quality (McCormick et al., 2005). Our study found that DVA was significantly correlated with SR at 40 and 80 dps. The result indicated that the dynamic visual performance under high velocity was affected by the retinal image quality. According to the theory of retinal smear, the extent of retinal image artifact increases as the velocity of the visual target increases (Geer and Robertson, 1993). The artifact is induced by the moving retinal image and the HOAs. Therefore, it can be inferred that the influence of HOAs also increases along with the moving speed of optotypes.

The multivariate linear regression analysis for DVA revealed that age was a significant determinant for DVA, and the older the age, the worse the DVA. The result was consistent with other previous studies (Ishigaki and Miyao, 1994; Lee et al., 2022). The senescence of the central nervous system was thought to be related to the decline in DVA (Ishigaki and Miyao, 1994). It should be noted that RMS_coma_ was not associated with 40 dps or 80 dps DVA in the multivariate regression model, which was inconsistent with the results of the univariate analysis. We further analyzed the correlation between age and aberrations. The results showed that the coma aberration was associated with age but not trefoil aberration. Previous studies also confirmed the positive correlation between corneal coma aberrations and age (Amano et al., 2004; Kiuchi et al., 2021). Thus, age may conceal the effect of RMS_coma_ on DVA, resulting in the disparity between multivariate and univariate analysis models.

Currently, cataract surgery has become a method to improve quality of life. Since dynamic vision is crucial in daily tasks, DVA is increasingly applied as an indicator to reflect functional vision in postoperative patients. The postoperative corneal HOAs cause visual disturbance that might affect dynamic vision, and the present research found that corneal coma and trefoil aberration had negative impacts on DVA. The outcome of the study helps clinical doctors understand the effect of corneal HOAs on DVA and guides communication with patients complaining of unsatisfactory dynamic visual experience after cataract surgeries. The research also provides a basis for advanced corneal HOAs-corrected IOL design to improve postoperative dynamic visual performance.

Certain limitations exist in the present study. First, we merely analyze the corneal HOAs. However, the intraocular HOAs may also influence dynamic visual performance. Second, the pupil diameter during the DVA test is not measured. Thus, the HOA profile during the DVA test could not be accurately speculated. Third, only one moving pattern is used in the present research. Different relationships might be found between HOAs and DVA with other moving patterns, including vertical and diagonal movements. Further studies are needed to evaluate the correlation between DVA and HOAs under different moving modes. Fourth, Wang et al. (2017) and Cione et al. (2023b) recommended performing a sample-size calculation to determine the minimum number of patients to be included. However, this is quite difficult for our study as there was no similar research before, which may cause potential statistical bias. Fifth, the present research includes some subjects with both eyes. The inter-eye correlation could affect the statistical significance when bilateral eyes are included in the same group (Hoffer et al., 2015; De Bernardo et al., 2020). Random choice of eyes in a single subject should be adopted in future studies. Sixth, patients with history of corneal refractive surgeries are excluded from our study as the change in the morphology of cornea can cause bias in the evaluation of both HOAs and prediction refractive error after cataract surgery, due to inaccurate measurement of anterior keratometry and the variation of keratometric index after refractive surgery. It is necessary to use specific methods of IOL power calculation in these types of eyes (Cione et al., 2023a). Further studies are needed to analyze the influence of corneal HOAs on DVA in cataract patients with history of corneal refractive surgeries.

In conclusion, the present research demonstrates that corneal HOAs negatively affect the high-speed DVA in age-related cataract patients after surgery. The vertical coma and RMS_coma_ are statistically correlated with 80 dps DVA. The vertical trefoil, RMS_trefoil_ and total RMS_HOA_ are statistically correlated with 40 and 80 dps DVA. The spherical aberrations are not associated with postoperative DVA. The present study might enable doctors to guide patients with various corneal HOAs on DVA-related tasks after cataract surgery.

## Data availability statement

The raw data supporting the conclusions of this article will be made available by the authors, without undue reservation.

## Ethics statement

The studies involving humans were approved by the Ethics Committee of Peking University Third Hospital (NO. M2023201). The studies were conducted in accordance with the local legislation and institutional requirements. The participants provided their written informed consent to participate in this study.

## Author contributions

TW: Conceptualization, Formal analysis, Investigation, Methodology, Visualization, Writing – original draft. YW: Conceptualization, Funding acquisition, Methodology, Project administration, Visualization, Writing – review and editing. YTL: Investigation, Project administration, Visualization, Writing – review and editing. YHL: Investigation, Software, Writing – review and editing. XJ: Conceptualization, Methodology, Project administration, Supervision, Writing – review and editing. XL: Conceptualization, Funding acquisition, Methodology, Project administration, Supervision, Writing – review and editing.

## References

[B1] Amano S. Amano Y. Yamagami S. Miyai T. Miyata K. Samejima T. (2004). Age-related changes in corneal and ocular higher-order wavefront aberrations. *Am. J. Ophthalmol.* 137 988–992. 15183781 10.1016/j.ajo.2004.01.005

[B2] Ao M. Li X. Huang C. Hou Z. Qiu W. Wang W. (2014). Significant improvement in dynamic visual acuity after cataract surgery: A promising potential parameter for functional vision. *PLoS One* 9:e115812. 10.1371/journal.pone.0115812 25541959 PMC4277412

[B3] Assi L. Chamseddine F. Ibrahim P. Sabbagh H. Rosman L. Congdon N. (2021). A Global assessment of eye health and quality of life: A systematic review of systematic reviews. *JAMA Ophthalmol.* 139 526–541. 33576772 10.1001/jamaophthalmol.2021.0146 PMC7881366

[B4] Atchison D. A. Guo H. Charman W. N. Fisher S. W. (2009). Blur limits for defocus, astigmatism and trefoil. *Vis. Res.* 49 2393–2403. 19631683 10.1016/j.visres.2009.07.009

[B5] Cione F. Gioia M. Pagliarulo S. (2023b). Bias That should be avoided to obtain a reliable study of IOL power calculation after myopic refractive surgery. *J. Refract Surg.* 39:68. 10.3928/1081597X-20221122-02 36630434

[B6] Cione F. De Bernardo M. Gioia M. Oliviero M. Santoro A. G. Caputo A. (2023a). A no-history multi-formula approach to improve the IOL power calculation after laser refractive surgery: Preliminary results. *J. Clin. Med.* 12:2890. 10.3390/jcm12082890 37109228 PMC10144756

[B7] De Bernardo M. Cione F. Rosa N. (2020). Methods for intraocular lens power calculation in cataract surgery after radial keratotomy. *Ophthalmology* 127:e87. 10.1016/j.ophtha.2019.08.019 31561878

[B8] Dexl A. K. Zaluski S. Rasp M. Grabner G. (2014). Visual performance after bilateral implantation of a new diffractive aspheric multifocal intraocular lens with a 3.5 D addition. *Eur. J. Ophthalmol.* 24 35–43. 10.5301/ejo.5000315 23787450

[B9] Fernández-Sánchez V. Ponce M. E. Lara F. Montés-Micó R. Castejón-Mochón J. F. López-Gil N. (2008). Effect of 3rd-order aberrations on human vision. *J. Cataract Refract. Surg.* 34 1339–1344. 18655985 10.1016/j.jcrs.2008.04.017

[B10] Geer I. Robertson K. M. (1993). Measurement of central and peripheral dynamic visual acuity thresholds during ocular pursuit of a moving target. *Optom. Vis. Sci.* 70 552–560. 10.1097/00006324-199307000-00006 8355967

[B11] Hashemi H. Pakzad R. Yekta A. Aghamirsalim M. Pakbin M. Ramin S. (2020). Global and regional prevalence of age-related cataract: A comprehensive systematic review and meta-analysis. *Eye* 34 1357–1370. 32055021 10.1038/s41433-020-0806-3 PMC7376226

[B12] Hoffer K. J. Aramberri J. Haigis W. Olsen T. Savini G. Shammas H. J. (2015). Protocols for studies of intraocular lens formula accuracy. *Am. J. Ophthalmol.* 160:403–5.e1. 26117311 10.1016/j.ajo.2015.05.029

[B13] Ishigaki H. Miyao M. (1994). Implications for dynamic visual acuity with changes in aged and sex. *Percept. Mot. Skills* 78 363–369. 10.2466/pms.1994.78.2.363 8022663

[B14] Ishiguro N. Horiguchi H. Katagiri S. Shiba T. Nakano T. (2022). Correlation between higher-order aberration and photophobia after cataract surgery. *PLoS One* 17:e0274705. 10.1371/journal.pone.0274705 36107829 PMC9477362

[B15] Jing D. Jiang X. Ren X. Su J. Wei S. Hao R. (2022). Change patterns in corneal intrinsic aberrations and nerve density after cataract surgery in patients with dry eye disease. *J. Clin. Med.* 11:5697. 10.3390/jcm11195697 36233565 PMC9572385

[B16] Ke S. Wan W. Li C. (2022). Comparisons of visual outcomes between bilateral implantation and mix-and-match implantation of three types intraocular lenses. *Int. Ophthalmol.* 43 1143–1152. 10.1007/s10792-022-02513-0 36125586

[B17] Kiuchi G. Hiraoka T. Ueno Y. Mihashi T. Oshika T. (2021). Influence of refractive status and age on corneal higher-order aberration. *Vis. Res.* 181 32–37. 33517073 10.1016/j.visres.2020.12.007

[B18] Lee J. S. Liu Y. H. Chen W. M. Lin K. K. Chang S. T. Lim A. Y. (2022). Association of sports vision with age, gender, and static visual acuity among nonathletic population. *Taiwan J. Ophthalmol.* 12 53–60. 10.4103/tjo.tjo_60_20 35399972 PMC8988984

[B19] Lombardo M. Lombardo G. (2010). Wave aberration of human eyes and new descriptors of image optical quality and visual performance. *J. Cataract Refract. Surg.* 36 313–331. 10.1016/j.jcrs.2009.09.026 20152616

[B20] Maloney R. K. Bogan S. J. Waring G. O. III (1993). Determination of corneal image-forming properties from corneal topography. *Am. J. Ophthalmol.* 115 31–41. 8420375 10.1016/s0002-9394(14)73521-4

[B21] McCormick G. J. Porter J. Cox I. G. MacRae S. (2005). Higher-order aberrations in eyes with irregular corneas after laser refractive surgery. *Ophthalmology* 112 1699–1709. 16095700 10.1016/j.ophtha.2005.04.022

[B22] Miháltz K. Vécsei-Marlovits P. V. (2021). The impact of visual axis position on the optical quality after implantation of multifocal intraocular lenses with different asphericity values. *Graefes Arch. Clin. Exp. Ophthalmol.* 259 673–683. 10.1007/s00417-020-05052-5 33471202

[B23] Nanavaty M. A. Spalton D. J. Marshall J. (2010). Effect of intraocular lens asphericity on vertical coma aberration. *J. Cataract Refract. Surg.* 36 215–221. 20152600 10.1016/j.jcrs.2009.08.024

[B24] Oliveira C. M. Ferreira A. Franco S. (2012). Wavefront analysis and Zernike polynomial decomposition for evaluation of corneal optical quality. *J. Cataract Refract. Surg.* 38 343–356. 10.1016/j.jcrs.2011.11.016 22176886

[B25] Ren X. Wang Y. Wang D. Wu B. Wu L. Xu Y. (2020). A novel standardized test system to evaluate dynamic visual acuity post trifocal or monofocal intraocular lens implantation: A multicenter study. *Eye* 34 2235–2241. 32024972 10.1038/s41433-020-0780-9 PMC7784919

[B26] Rocha K. M. Nosé W. Bottós K. Bottós J. Morimoto L. Soriano E. (2007). Higher-order aberrations of age-related cataract. *J. Cataract Refract. Surg.* 33 1442–1446. 17662439 10.1016/j.jcrs.2007.03.059

[B27] Schrecker J. Feith A. Langenbucher A. (2014). Comparison of additional pseudophakic multifocal lenses and multifocal intraocular lens in the capsular bag. *Br. J. Ophthalmol.* 98 915–919. 10.1136/bjophthalmol-2013-304591 24554737

[B28] Sinjab M. M. (2021). *Corneal tomography in clinical practice (Pentacam system): Basics and clinical interpretation*, 4th Edn, Vol. 1. London: JP Medical Publishers, 28.

[B29] Steinberg E. P. Tielsch J. M. Schein O. D. Javitt J. C. Sharkey P. Cassard S. D. (1994). The VF-14. An index of functional impairment in patients with cataract. *Arch. Ophthalmol.* 112 630–638. 8185520 10.1001/archopht.1994.01090170074026

[B30] Tabernero J. Piers P. Artal P. (2007). Intraocular lens to correct corneal coma. *Opt. Lett.* 32 406–408. 17356668 10.1364/ol.32.000406

[B31] Wang L. Koch D. D. Hill W. Abulafia A. (2017). Pursuing perfection in intraocular lens calculations: III. Criteria for analyzing outcomes. *J. Cataract Refract. Surg.* 43 999–1002. 10.1016/j.jcrs.2017.08.003 28917430

[B32] Wang Y. Guo Y. Li Y. Zhang Y. Yuan Y. Wu T. (2023). The impact of different corneal refractive surgeries on binocular dynamic visual acuity. *Front. Neurosci.* 17:1142339. 10.3389/fnins.2023.1142339 36937680 PMC10022881

[B33] Wu T. Wang Y. Wei S. Guo Y. Li X. (2022). Developing dynamic defocus curve for evaluating dynamic vision accommodative function. *BMC Ophthalmol.* 22:106. 10.1186/s12886-022-02335-9 35248018 PMC8898511

[B34] Wu T. Wang Y. Yu J. Ren X. Li Y. Qiu W. (2023). Comparison of dynamic defocus curve on cataract patients implanting extended depth of focus and monofocal intraocular lens. *Eye Vis.* 10:5. 10.1186/s40662-022-00323-0 36721199 PMC9890684

[B35] Xi L. (2020). Wavefront properties of the anterior and posterior corneal surface after transepithelial photorefractive keratectomy in myopia. *Exp. Ther. Med.* 19 1183–1188. 10.3892/etm.2019.8338 32010287 PMC6966128

[B36] Xu Y. Deng J. Zhang B. Xu X. Cheng T. Wang J. (2022). Higher-order aberrations and their association with axial elongation in highly myopic children and adolescents. *Br. J. Ophthalmol.* 107 862–868. 10.1136/bjophthalmol-2021-319769 35027355 PMC10314045

[B37] Yamaguchi T. Negishi K. Ohnuma K. Tsubota K. (2011). Correlation between contrast sensitivity and higher-order aberration based on pupil diameter after cataract surgery. *Clin. Ophthalmol.* 5 1701–1707. 10.2147/OPTH.S21819 22205829 PMC3245194

[B38] Ye H. Zhang K. Yang J. Lu Y. (2014). Changes of corneal higher-order aberrations after cataract surgery. *Optom. Vis. Sci.* 91 1244–1250. 25105685 10.1097/OPX.0000000000000362

