# Supplementary material for: The impact of corneal higher-order aberrations on dynamic visual acuity post cataract surgery
Source: Front Neurosci. 2024 May 13;18:1321423. doi: 10.3389/fnins.2024.1321423 (PMC11128552; doi:10.3389/fnins.2024.1321423)
Supplement: Supplementary file 1 [file Table_1.docx]

**Supplementary Table. Correlation analysis between postoperative dynamic visual acuity and corneal higher-order aberrations represented by Zernike polynomial coefficients.**

| **Parameters** | **DVA (20 dps)** | | **DVA (40 dps)** | | **DVA (80 dps)** | |
| --- | --- | --- | --- | --- | --- | --- |
|  | **R** | **P** | **R** | **P** | **R** | **P** |
| $Z_{4}^{0}$ (4mm) | -0.127 | 0.406 | 0.047 | 0.760 | 0.119 | 0.436 |
| $Z_{4}^{0}$ (5mm) | -0.139 | 0.364 | -0.034 | 0.823 | 0.117 | 0.446 |
| $Z_{4}^{0}$ (6mm) | -0.064 | 0.677 | -0.087 | 0.570 | 0.088 | 0.566 |
| $Z_{4}^{0}$ (7mm) | -0.031 | 0.841 | -0.099 | 0.518 | 0.028 | 0.858 |
| $Z_{6}^{0}$ (4mm) | -0.077 | 0.616 | -0.133 | 0.383 | -0.020 | 0.896 |
| $Z_{6}^{0}$ (5mm) | 0.101 | 0.508 | -0.073 | 0.632 | 0.031 | 0.839 |
| $Z_{6}^{0}$ (6mm) | 0.234 | 0.121 | 0.030 | 0.844 | -0.019 | 0.904 |
| $Z_{6}^{0}$ (7mm) | 0.071 | 0.645 | -0.083 | 0.589 | -0.171 | 0.261 |
| $Z_{3}^{-1}$ (4mm) | 0.142 | 0.353 | 0.150 | 0.324 | 0.328 | **0.028^*^** |
| $Z_{3}^{-1}$ (5mm) | 0.206 | 0.174 | 0.226 | 0.135 | 0.354 | **0.017^*^** |
| $Z_{3}^{-1}$ (6mm) | 0.253 | 0.093 | 0.235 | 0.120 | 0.340 | **0.022^*^** |
| $Z_{3}^{-1}$ (7mm) | 0.265 | 0.078 | 0.215 | 0.156 | 0.340 | **0.022^*^** |
| $Z_{3}^{1}$ (4mm) | -0.131 | 0.391 | -0.108 | 0.480 | -0.126 | 0.409 |
| $Z_{3}^{1}$ (5mm) | -0.073 | 0.633 | 0.034 | 0.825 | 0.008 | 0.957 |
| $Z_{3}^{1}$ (6mm) | -0.069 | 0.653 | 0.122 | 0.426 | 0.080 | 0.602 |
| $Z_{3}^{1}$ (7mm) | -0.147 | 0.334 | 0.048 | 0.752 | 0.007 | 0.963 |
| $Z_{5}^{-1}$ (4mm) | -0.023 | 0.882 | 0.125 | 0.415 | -0.005 | 0.972 |
| $Z_{5}^{-1}$ (5mm) | 0.060 | 0.695 | 0.024 | 0.877 | -0.122 | 0.423 |
| $Z_{5}^{-1}$ (6mm) | 0.058 | 0.703 | -0.030 | 0.846 | -0.178 | 0.242 |
| $Z_{5}^{-1}$ (7mm) | -0.089 | 0.561 | -0.188 | 0.217 | -0.281 | 0.062 |
| $Z_{5}^{1}$ (4mm) | -0.035 | 0.820 | 0.168 | 0.271 | 0.124 | 0.417 |
| $Z_{5}^{1}$ (5mm) | 0.051 | 0.740 | 0.130 | 0.395 | 0.147 | 0.335 |
| $Z_{5}^{1}$ (6mm) | 0.069 | 0.653 | 0.117 | 0.442 | 0.092 | 0.548 |
| $Z_{5}^{1}$ (7mm) | -0.016 | 0.915 | 0.125 | 0.414 | 0.132 | 0.387 |
| $Z_{3}^{-3}$ (4mm) | 0.136 | 0.375 | -0.298 | **0.047*** | -0.240 | 0.113 |
| $Z_{3}^{-3}$ (5mm) | 0.006 | 0.970 | -0.382 | **0.010**** | -0.326 | **0.029*** |
| $Z_{3}^{-3}$ (6mm) | -0.047 | 0.760 | -0.365 | **0.014*** | -0.303 | **0.043*** |
| $Z_{3}^{-3}$ (7mm) | -0.105 | 0.493 | -0.363 | **0.014*** | -0.319 | **0.033*** |
| $Z_{3}^{3}$ (4mm) | -0.066 | 0.665 | 0.042 | 0.782 | -0.129 | 0.398 |
| $Z_{3}^{3}$ (5mm) | -0.057 | 0.712 | 0.046 | 0.763 | -0.180 | 0.238 |
| $Z_{3}^{3}$ (6mm) | -0.071 | 0.642 | 0.078 | 0.612 | -0.191 | 0.209 |
| $Z_{3}^{3}$ (7mm) | -0.040 | 0.792 | 0.079 | 0.607 | -0.191 | 0.209 |
| $Z_{5}^{-3}$ (4mm) | -0.149 | 0.330 | -0.163 | 0.285 | -0.284 | 0.059 |
| $Z_{5}^{-3}$ (5mm) | -0.195 | 0.198 | 0.034 | 0.827 | -0.060 | 0.694 |
| $Z_{5}^{-3}$ (6mm) | -0.312 | **0.037^*^** | -0.035 | 0.822 | 0.022 | 0.888 |
| $Z_{5}^{-3}$ (7mm) | -0.271 | 0.072 | 0.140 | 0.360 | 0.145 | 0.342 |
| $Z_{5}^{3}$ (4mm) | 0.020 | 0.897 | -0.056 | 0.716 | -0.125 | 0.414 |
| $Z_{5}^{3}$ (5mm) | 0.097 | 0.528 | 0.014 | 0.928 | -0.112 | 0.466 |
| $Z_{5}^{3}$ (6mm) | 0.184 | 0.226 | 0.158 | 0.299 | -0.021 | 0.889 |
| $Z_{5}^{3}$ (7mm) | 0.172 | 0.258 | 0.129 | 0.399 | -0.020 | 0.895 |

Dps = degrees per second, DVA = dynamic visual acuity, P = probability value, R= correlation coefficient

The boldface indicates statistical significance

* indicates statistical significance P ≤ 0.05

** indicates statistical significance P ≤ 0.01
